# The Sleep of Shift Workers in a Remote Mining Operation: Methodology for a Randomized Control Trial to Determine Evidence-Based Interventions

**DOI:** 10.3389/fnins.2020.579668

**Published:** 2021-01-07

**Authors:** Gemma Maisey, Marcus Cattani, Amanda Devine, Johnny Lo, Ian C. Dunican

**Affiliations:** ^1^School of Medical and Health Science, Edith Cowan University, Perth, WA, Australia; ^2^School of Science, Edith Cowan University, Perth, WA, Australia

**Keywords:** shiftwork, fatigue, sleep disorders, education, wrist-activity monitors, biomathematical modeling, sleep loss

## Abstract

Shiftwork may adversely impact an individual’s sleep-wake patterns and result in sleep loss (<6 h. following night shift), due to the circadian misalignment and the design of rosters and shifts. Within a mining operation, this sleep loss may have significant consequences due to fatigue, including an increased risk of accidents and chronic health conditions. This study aims to (i) determine the efficacy of an intervention that comprises a sleep education program and biofeedback through a smartphone app on sleep quality, quantity, and alertness (ii) determine the prevalence of risk for a potential sleep disorder, and (iii) quantify and describe the sleep habits and behaviors of shift workers in a remote mining operation. This study consists of a randomized controlled trial whereby eighty-eight shift workers within a remote mining operation are randomized to a control group or one of three different treatment groups that are: (i) a sleep education program, (ii) biofeedback on sleep through a smartphone app, or (iii) a sleep education program and biofeedback on sleep through a smartphone app. This study utilizes wrist-activity monitors, biomathematical modeling, and a survey instrument to obtain data on sleep quantity, quality, and alertness. A variety of statistical methods will determine the prevalence of risk for a potential sleep disorder and associations with body mass index, alcohol, and caffeine consumption. A generalized linear mixed model will examine the dependent sleep variables assessed at baseline and post-intervention for the control group and intervention groups, as well as within and between groups to determine changes. The findings from this study will contribute to the current understanding of sleep and alertness behaviors, and sleep problems and disorders amongst shift workers. Importantly, the results may inform fatigue policy and practice on interventions to manage fatigue risk within the mining industry. This study protocol may have a broader application in other shiftwork industries, including oil and gas, aviation, rail, and healthcare.

## Introduction

In today’s industrial society many industries operate 24 h, 7 days a week, 365 days (24/7/365) a year to maximize their return on investment by utilizing assets and people to meet customer needs. One such sector is mining, which involves the extraction of minerals (e.g., iron ore, gold, coal) from the earth, that are then processed and sold for use in industries including construction and manufacturing (Healey, 2012).

To enable mining companies to operate 24/7/365, individuals are required to work outside of the standard daylight hours including, early mornings (e.g., 06:00–14:00) evenings (e.g., 14:00–23:00), or during the night (e.g., 23:00–06:00). These business requirements and shiftwork patterns may lead to what is commonly known as “fatigue.” While several definitions exist for fatigue, in the context of our study, fatigue is the result of sleep loss (<7 h) and/or being awake during an adverse circadian phase, thereby potentially affecting alertness (Lerman et al., 2012; Phillips, 2015).

Regulation of the human sleep-wake cycle is attributed to two mechanisms (i) the circadian system and (ii) the homeostatic drive. The circadian system refers to the body’s biological clock known as the suprachiasmatic nuclei, which regulates sleep-wake patterns with a 24 h daily cycle. The homeostatic drive refers to the pressure for sleep that increases over time due to the hours of wakefulness (Borbély et al., 2016). Shift workers who work nights, often experience a circadian misalignment as they are required to modify their sleep-wake patterns by sleeping during the day when their circadian drive for alertness peaks (Kazemi et al., 2016). Furthermore, the design of roster schedules and shifts may determine and potentially limit an individual’s opportunity to sleep. Design elements may include long shift durations of >12 h, early shift start times (before 06:00), and backward rotating rosters (night to day shifts) (Akerstedt and Wright, 2009). The roster schedule design may also interfere with sleep regularity in shift workers contributing to intra-individual variability in the timing of sleep onset and wake time when on workdays compared to days off (Murray et al., 2019).

The National Sleep Foundation recommends 7–9 h of sleep duration per night for a healthy adult, and less than this may be considered to be sleep loss (Hirshkowitz et al., 2015). Amongst the general population, approximately 1 in 3 adults experience sleep loss (Yong et al., 2016). It is estimated that half of these sleep problems are attributed to the presence of sleep disorders while the remainder may be due to individual behaviors that limit the opportunity for sleep (Hillman and Lack, 2013). Among the most common sleep disorders are obstructive sleep apnea (OSA), restless legs syndrome, and insomnia (Adams et al., 2017). Also, the prevalence of shiftwork disorder in shiftwork populations has been reported as 23–63% (Vanttola et al., 2019). The symptoms of shiftwork disorder are similar to insomnia, including difficulty falling asleep and staying asleep, however, they are associated with an individual’s roster cycle (Booker et al., 2018).

In remote mining companies, the prevalence of sleep loss may be higher than the general population due to irregularities of the sleep-wake cycle resulting from the roster design, long work hours, job demands, stress, the presence of sleep disorders, individual behaviors, and the requirement to reside in camp accommodation during work periods. This sleep loss may result in health and safety consequences for shift workers (Barnes and Watson, 2019). Sleep loss has been associated with a significant increase in the risk of developing chronic health conditions such as obesity, diabetes, cancer, cardiovascular disease, and mental health disorders (James et al., 2017). Furthermore, it may increase the risk of accidents by impairing human alertness, which is characterized by an increase in reaction time, inability to make decisions, and poor concentration (Halvani, 2009; Chellappa et al., 2019). An analysis of reported injury rates indicated that the risk of injury increases by 28% on night shifts compared to the morning shifts, increases over successive night shifts (36% higher risk on the fourth night), and increases with hours of duty with an estimated 27% increased risk for 12 h shifts compared to 8 h shifts (Folkard and Lombardi, 2006). In Australia, the reported injury rates for shift workers are over double that of non-shift workers (Safe Work Australia, 2016). Furthermore, a study into the prevalence and social impacts of sleep problems in Australia found that 29% of adults reported making errors at work due to sleepiness or sleep problems (Adams et al., 2017).

Given the potential consequences of fatigue in a shiftwork mining operation, an opportunity exists to develop and deploy evidence-based interventions. Such interventions, including a sleep education program and biofeedback on sleep through a smartphone application (app), may reduce fatigue risk through supporting individual behavior change to improve sleep quantity and quality. Currently, no studies exist evaluating the efficacy of these interventions in a mining operation.

Sleep education programs for shift workers commonly include information on circadian physiology; the link between shiftwork and inadequate sleep; lifestyle and behavior advice relating to exercise, diet, alcohol, and caffeine; environmental factors that influence sleep such as light, noise, and temperature; how to recognize and manage fatigue at work; and sleep disorders (Davy, 2014). Studies on the effect of such programs on the safety, performance, fatigue, and sleep quality of shift workers have found favorable outcomes (*n* = 13) in health care, aviation, emergency services, and other similar industries. Increases in awareness and knowledge of fatigue following participation in sleep education have been associated with improved sleep and performance, including reduced errors and enhanced reaction times (Barger et al., 2018). However, the quality of evidence for these studies was deemed low due to a lack of field-based randomized control trials (RCT) (Barger et al., 2018). Furthermore, a limitation common to these studies is the subjective assessment of sleep using questionnaires to assess self-reported measures. Self-reported sleep data may contain some degree of response bias whereby individuals either overestimate or underestimate their sleep, depending on the question (Croy et al., 2017).

The use of smartphone apps to deliver objective biofeedback may be an effective mechanism for individuals to identify and understand their sleep behaviors (Liang et al., 2016). Wrist-activity monitors have the capability of connecting to a smartphone app and can provide instant feedback on sleep. Regular monitoring of these behaviors allows individuals to make lifestyle choices pro-actively and potentially change behaviors that promote sleep (Shin et al., 2019). To date, a scarcity of research exists with only five studies that have evaluated wrist-activity monitor or smartphone apps that provide biofeedback on sleep as an intervention to improve sleep outcomes, all with variable results. Two of these studies were conducted in the general population, one within a workplace setting, one with college students, and one with insomnia patients (Baron et al., 2018). No studies have been conducted on shift workers or mining operations.

Therefore our study involves a partnership with a remote mining operation in regional Western Australia (WA) that operates on a 24/7/365 days a year business cycle requiring shift workers to work seven-day shifts, followed by seven-night shifts of a 12 h duration, with seven days off before repeating the rotation (7 days/7 nights/7 off). We hypothesize that the provision of sleep education, combined with biofeedback on sleep through a smartphone app, will result in improved sleep quality, quantity, and alertness outcomes for shift workers in mining operations. Our RCT aims to (i) determine the efficacy of an intervention that comprises a sleep education program and biofeedback through a smartphone app on sleep quality, quantity, and alertness; (ii) determine the prevalence of risk for a potential sleep disorder; and (iii) quantify and describe the sleep habits and behaviors of shift workers in a remote mining operation.

## Materials and Methods

### Experimental Design Overview

**FIGURE 1:**
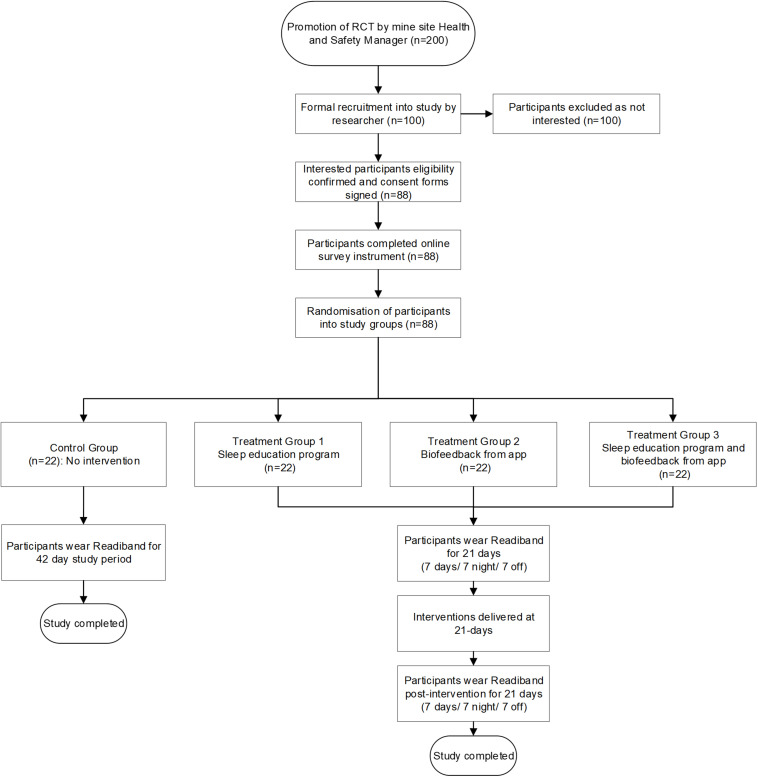
RCT design and flow of participants through the study.

Our RCT study design includes participants being randomly allocated to a control group or one of three different treatment groups that are: (1) sleep education, (2) biofeedback on sleep through a smartphone app, or (3) sleep education and biofeedback on sleep through a smartphone app. This study will examine sleep quantity, quality, and alertness outcome measures at baseline and post-intervention. All participants will wear a wrist-activity monitor to obtain objective sleep measures for the 42-day study period, during which they will work two roster cycles (7 days/7 nights/7 off; 7 days/7 nights/7 off). Participants randomized to the treatment groups will receive intervention at day 21. Control group participants will not receive any intervention allowing for comparison with the treatment groups. Figure 1 displays the RCT design and flow of participants through the study.

### Setting

This study will take place within a remote mine site operation, located in the North West of WA. The core business of this mine site is the extraction of iron ore from the outermost layer of the earth, for the production of steel that is used in the construction and manufacturing industries. The mining process commences with exploration to identify commercially viable iron ore deposits. A drill and blast team then drill the portion of the ore body to be removed, load the drill holes with explosives and blast the rock breaking it for excavation. The fractured ore is loaded into haul trucks, and the product that does not meet quality grade is deposited at a waste dump and used for mine rehabilitation upon mine closure. The quality product known as the saleable ore product is delivered to a plant and passed through a crushing, screening, and blending process (Lu, 2015). The mine site mobile maintenance team is responsible for the maintenance of essential equipment (including drills, haul trucks, loaders, and dozers) required to meet production targets. This study will recruit participants from the mine site mobile maintenance and drill and blast teams working a 7 days/7 nights/7 off roster cycle. The remote location of the mine site requires participants to travel from the state capital on their first-day shift, returning at the end of their last night shift of the roster cycle. Participants resided in their room within the mine camp accommodation during the roster cycle, commuting 25 min by bus to and from their work location for day shift (start time 05:30; end time 17:30) and night shift (start time 17:30; end time 5:30).

### Recruitment

The mine site Health and Safety Manager will initiate the recruitment process through a short presentation to the mobile maintenance and drill and blast teams (*n* = 200) and email an information sheet to interested participants (*n* = 100). Following this, the researcher will travel twice to the mine site over one week to enroll participants into the study. The enrolment process involves participants (i) attending an information session, (ii) completing a screening questionnaire to confirm their eligibility, and (iii) providing their informed consent to participate. Participants are eligible for inclusion if they work 7 days/7 nights/7 off roster cycle, day shift (start time 05:30; end time 17:30), night shift (start time 17:30; end time 5:30), and have no leave planned for the 42-day study duration that includes two roster cycles. No exclusion criteria are defined to achieve a representative sample of shift workers in a mining operation. Eighty-eight (*n* = 88) participants will be enrolled in the study.

### Sample Size

A sample size calculation was conducted using G^∗^Power version 3.0.10. A minimum total sample of 68 participants is required to detect a medium Group x Time interaction effect (Cohen’s *f*^2^ = 0.25) at 80% power and 1% level of significance (α) based on a generalized linear mixed model design with four groups and two time-points. The specified α at 1%, instead of the usual 5%, was to account for the modeling of multiple outcomes, i.e., sleep onset latency, sleep duration, wake after sleep onset, fragmentation index, and sleep efficiency. An estimated attrition rate of 20% is assumed. Therefore, a total of 88 participants will be recruited, allowing for 22 participants in the control group and each of the three treatment groups.

### Randomization

The randomization of participants to the groups will occur directly after enrolment into the study. Allocation to the control or treatment groups will be through the random selection of a wrist-activity monitor from a concealed box. Each monitor will be preassigned (by the researcher) to the control group or a treatment group using a color-coding system: gold (control group), white (treatment group 1), pink (treatment group 2), and green (treatment group 3). Only the researcher will know the color corresponding to each group, and the monitor selection process will be managed by an independent employee of the mine site to reduce any potential bias. Each participant will select a monitor, and their name recorded against the monitor’s serial number. The researchers, employees of the mine site, nor the participants will have the knowledge to which group a participant will be assigned at the time of randomization. Participants will be informed of their assigned group (control or treatment 1–3) after randomization. The researcher will not interact directly with participants from this point in time to the intervention date.

### Conditions

#### Treatment Groups

Treatment group participants will wear a wrist-activity monitor for the 42-day study and will be assigned to one of three interventions received on day 21, as follows:

(1) *Sleep education program:* Participants completed a 2-h face-to-face education program, delivered on-site by the researcher, while participants are at work. The education program aims to empower participants with the knowledge and practical strategies to improve their sleep and includes information on sleep physiology, how shiftwork impacts sleep, common sleep problems and disorders, and sleep hygiene practices. Upon completion of the program, participants will complete a short 12 item quiz to assess their knowledge of good sleep hygiene practices (see Supplementary Document 1).
(2) *Biofeedback on sleep through a smartphone app:* Participants receive biofeedback through a smartphone app on sleep quality, quantity, and alertness. Participants will receive an email with details of the smartphone app to download and an invitation to attend a 15-min information session to assist with the smartphone app download and use. The app will provide participants with; their current, predicted alertness (24 h prediction) and trends, their most recent sleep, and their sleep over the last seven days. Sleep measures provided by the smartphone app include sleep duration, time at sleep onset, time at wake, sleep efficiency, and wake after sleep onset. Participants will be encouraged to sync their wrist-activity monitor daily with their smartphone app and use this information to make behavior changes to improve their sleep and alertness. On day 21 of the study, the researcher will confirm all participants assigned to treatment groups 2 and 3 have activated their smartphone app account.
(3) *Sleep education program and biofeedback on sleep through a smartphone app:* Participants complete the same 2 h face-to-face education program outline in intervention one combined with biofeedback on sleep from a smartphone app as outlined in intervention two.

Treatment group 1 will receive a sleep education program, treatment group 2 biofeedback on sleep through a smartphone app, and treatment group 3 a sleep education program and biofeedback on sleep through a smartphone app.

#### Control Group

The control group participants will wear a wrist-activity monitor for the 42-day study and not receive any intervention. The inclusion of a control group will allow control group outcomes to be compared with the treatment group outcomes, increasing the confidence that any changes observed post-intervention are attributable to the intervention.

### Measures

#### Sleep Quantity and Quality

Participants will wear a Readiband^TM^ version 5 (Readiband^TM^, Fatigue Science Inc., Canada), wrist-activity monitor, 24 h, 7 days a week for the 42-day duration of the study to obtain objective sleep measures at baseline (day 0–21) and post-intervention (day 22–42). The Readiband^TM^ contains a tri-axial accelerometer that records the frequency and intensity of limb movement that can be converted to sleep-wake periods using an automated proprietary scoring algorithm contained within the Readiband Sync^TM^ software (Russell et al., 2000). The Readiband^TM^ has been validated against polysomnography (PSG), the gold standard for measuring sleep (Marino et al., 2013), and found to have an overall accuracy of detecting sleep-wake periods of 93% (Russell et al., 2000). This monitor has also produced comparable results (in-laboratory and in a home environment) to another validated wrist-activity monitor, the ActiGraph^TM^ (Dunican et al., 2018), and is approved by the United States Food and Drug Administration as a device to measure sleep and activity (Food and Drug Administration, 2011). The Readiband^TM^ provides a practical alternative to PSG for this field study, as it is unobtrusive, cost-effective, and can be worn over multiple weeks (Smith et al., 2018). The devices will be fitted with a quick-release strap for safety purposes making the Readiband^TM^ suitable to wear during work. Data will be downloaded weekly from the Readiband^TM^ using the Readiband Sync^TM^ software. In the context of this study, sleep quantity will be measured by sleep duration, which is derived from the number of minutes from time at sleep onset to time at wake, minus wake after sleep onset. Sleep duration of ≥7 h sleep per day/night will be considered optimal (Hirshkowitz et al., 2015). Sleep quality will be measured by sleep efficiency, which is derived from sleep duration divided by time in bed multiplied by 100. Sleep efficiency of ≥85% will be considered optimal (Ohayon et al., 2017). Individual alertness will be measured by the Readiband^TM^ app that utilizes the Sleep, Activity, Fatigue, and Task Effectiveness (SAFTE^TM^) algorithm. This app uses prior measured sleep-wake periods to predict alertness continuously. An alertness score of ≥80% will be considered optimal (Hursh et al., 2004b). The SAFTE^TM^ model is explained in more detail in section “Biomathematical Modeling” of this paper. Table 1 provides a summary of derived and direct measures from the Readiband^TM^ used to determine sleep quantity, quality, and alertness.

**TABLE 1:** Summary of derived and direct sleep measures from the Readiband^TM^ (Fatigue Science, Canada) device.

Sleep Measures	Units	Measurement	Description
Sleep Onset Latency	Minutes	Derived	Number of minutes from Time at Lights Out to Time at Sleep Onset.
Time at Sleep Onset	Time of day	Directly measured	Time of day when the first epoch of sleep occurs between Time at Lights Out and Time at Wake.
Time at Sleep Onset Variance	Minutes	Derived	The degree to which daily time at sleep onset differs from the mean time at sleep onset for an individual.
Sleep Duration	Minutes	Derived	Number of minutes from Time at Sleep Onset to Time at Wake, minus number of minutes awake (WASO).
Wake After Sleep Onset	Minutes	Directly measured	Number of minutes awake after Time at Sleep Onset.
Fragmentation Index	Frequency	Directly measured	Number of awakenings between Time at Sleep Onset and Time at Wake.
Time at Wake	hh:mm	Directly measured	Time that wake occurs with no further sleep duration.
Time at Wake Variance	Minutes	Derived	The degree to which daily time at wake differs from the mean time at wake for an individual.
Time in Bed	Minutes	Derived	The total time spent in bed, from Time at Lights Out to Time at Wake.
Sleep Efficiency	Percentage	Derived	Sleep Duration divided by Time in Bed multiplied by 100.
Alertness	Percentage	SAFTE^TM^	Measure of alertness calculated using the biomathematical model known as SAFTE^TM^ (Sleep, Activity, Fatigue, and Task Effectiveness) algorithm.

*This table is based upon Dunican et al. Sleep and Biological Rhythms, 2018.*

*Notes: This table provides a summary of the 11 derived and direct sleep measures from the Readiband^TM^ for analysis. A measure is classified as derived if it is a calculation of measures that are directly extracted from the Readiband.*

#### Biomathematical Modeling

The Sleep, Activity, Fatigue, and Task Effectiveness biomathematical model will be used to predict alertness across the roster schedule. The sleep-wake data inputs for the model will include sleep duration, time at sleep onset, wake after sleep onset, and time at wake as measured by the Readiband^TM^.

High-risk industries, including aviation, rail, and maritime, have widely used biomathematical modeling as a roster scheduling tool to reduce the fatigue-related accident risk associated with work-rest and sleep-wake patterns (James et al., 2018). The SAFTE^TM^ algorithm is a three-process model that incorporates the function of the homeostatic drive, the circadian system, and sleep inertia (the delay after awakening from sleep, before expected levels of alertness resume). A validation study of this model in the rail transportation industry, involving an examination of 1400 accidents found a positive association between predicted alertness, as measured by the SAFTE^TM^ model, and the risk of accidents ascribed to human factors (Hursh et al., 2006). A further validation study in the aviation industry found significant correlations between predicted alertness by the SAFTE^TM^ model and actual alertness, reaction times, and lapses measured by psychomotor vigilance tests (Roma et al., 2012). Psychomotor vigilance tests have been widely used in laboratory and field studies to determine the effects of fatigue on reaction times during sustained vigilance performance (Arsintescu et al., 2019).

**FIGURE 2:**
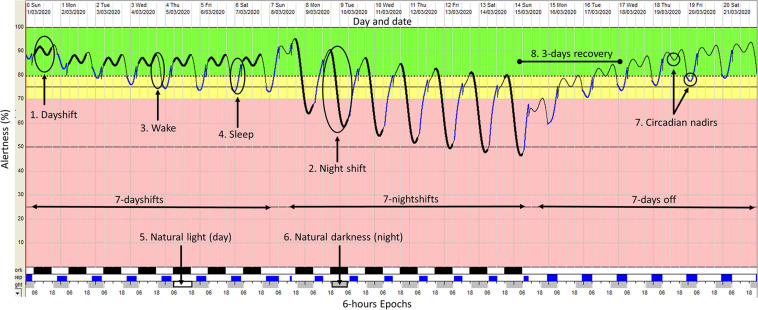
An example of the graphical output from the FAST^TM^ software. This FAST output depicts a 7 days/7 nights/7 days off roster cycle with sleep-wake inputs based on industry research (6.5h sleep when working dayshift; 5.5 h sleep when working night shift; 7 h sleep on days off) (Kecklund and Axelsson, 2016). Alertness is depicted by the oscillating colored line running through the center from left to right. The y-axis displays the alertness value as a percentage (1–100%) by time across the *x*-axis (6-hour epoch). The thick black line represents periods at work (dayshift or nightshift) (points 1 and 2), the thin black line represents periods of wake with no work and no sleep (point 3) and the blue line represents periods of sleep (point 4). The light gray and white blocks across the *x*-axis represent periods of natural light (daytime) (point 5) and natural darkness (night time) (point 6). The oscillating alertness line shows two circadian nadirs within every 24-hour (point 7). The first nadir occurs at night time (03:00–06:00) and the second during daytime (13:00–15:00). In this example, working a roster cycle that includes 7-day shifts followed by seven-night shifts, can take a shift worker 3 days to recover to optimal alertness levels (>80%) (point 8).

The Fatigue Avoidance Scheduling Tool (FAST^TM^), is the software interface for the SAFTE^TM^ model that provides the schedule inputs and predictions (Hursh et al., 2004a). The FAST^TM^ software produces a visual graphic that predicts alertness on a scale of 0–100% across time and displays the time spent below an adjustable fatigue risk criterion line. Figure 2 provides an example of the graphical output from FAST^TM^.

#### Survey Instrument

An online survey instrument will be administered using Qualtrics^TM^ directly proceeding enrolment of all participants to obtain demographic and anthropometric information and determine the potential prevalence of risk for a sleep disorder. The survey instrument includes scientifically validated sleep and alcohol consumption questionnaires to assess and measure the following:

##### Anthropometry

These measures will be self-reported and included height (m) and weight (kg). Body mass index (BMI) will then be calculated (kg/m^2^) and scored as either underweight (<18.5), healthy weight (18.5–25), overweight (25–30), or obese (≥30).

##### Demographics

A series of questions related to age, gender, place of residence, and educational attainment.

##### Obstructive sleep apnea

The Berlin Questionnaire will assess an individual’s risk of OSA. This tool consists of 11 questions on risk factors for OSA, including snoring, fatigue, and obesity. The questions are grouped into three categories and assessed as either positive or negative. This resultant binary outcome establishes either a Low-risk (<2 positives) or High-risk (≥2 positives) of OSA (Enciso and Clark, 2011).

##### Sleep quality

The Pittsburgh Sleep Quality Index will assess an individual’s sleep quality. This tool consists of 19 questions that examine the subjective aspects of sleep quality over one month. The questions are grouped into seven component scores that are summed to give a final score between 0 and 21. Higher scores indicate poor sleep quality (Buysse et al., 1989).

##### Daytime sleepiness

The Epworth Scale of Sleepiness will assess an individual’s likelihood of dozing off or falling asleep during everyday situations through a series of eight questions. A final score between 0 and 24 is achieved and assigned an outcome of lower normal (0–5), higher normal (6–10), mild excessive (11–12), moderate excessive (13–15), or severe excessive daytime sleepiness (16–24) (Doneh, 2015).

##### Insomnia

The Insomnia Severity Index will assess an individual’s risk of insomnia. This tool consists of seven questions assessing the severity of sleep-onset and sleep maintenance (rated on a 0–4 scale). A final score between 0 and 28 is achieved and assigned an outcome of no clinically significant insomnia (0–7), subthreshold insomnia (8–14), clinical insomnia moderate severity (15–21), or clinical insomnia severe (22–28) (Bastien et al., 2001).

##### Shiftwork disorder

The Shiftwork Disorder Screening Questionnaire will assess an individual’s risk of shiftwork disorder. This tool consists of four questions relating to excessive daytime sleepiness and/or insomnia due to a shiftwork cycle. The scoring involves multiple calculations whereby each question is multiplied by a classification function coefficient and the constant added. The score in the high-risk column score is compared with the low-risk column score, with the highest score indicating the individuals’ risk (Barger et al., 2012).

##### Alcohol use

The Alcohol Use Disorders Identification Test (AUDIT) developed by the World Health Organization will screen for excessive alcohol consumption. The test consists of 10 questions with each response scored 0–4. The total summed score indicates either low risk (≤7), hazardous or harmful alcohol consumption (≥8), or alcohol dependence or at risk of alcohol dependence (≥20) (Thomas et al., 2001).

### Dissemination of Participant Results

Participants will receive their sleep and alertness reports via an online account. Participants identified as obese, at risk of hazardous and/or harmful alcohol consumption or alcohol dependence, or risk for sleep disorders will be emailed their survey results and a recommendation to contact their General Practitioner for further review and advice. It must be noted that individual results will not be provided to the management of the mining operation.

### Statistical Analysis

Statistical analysis will be performed using SPSS v26 (IBM Corp., 2019). The prevalence of sleep disorders amongst the sample will be reported using descriptive statistics including frequencies, percentages, means, and standard deviations (normally distributed data) and median and interquartile range (non-normally distributed data). Multiple regression will be used to determine the relationship between insomnia and daytime sleepiness with age, gender, BMI score, alcohol consumption, caffeine consumption, smoking, non-prescribed, and prescribed medication use. Logistic regression with a forward stepwise variable selection will explore the relationship between OSA with shiftwork disorder outcomes, age, gender, BMI score, alcohol consumption, caffeine consumption, smoking, non-prescribed, and prescribed medication use; odds ratios (OR) and their 95% confidence interval (CI) will be reported. The effect of sleep education and biofeedback through a smartphone app on sleep variables including, sleep onset latency, time at sleep onset, sleep duration, wake after sleep onset, fragmentation index, time in bed, sleep efficiency, and time at wake will be tested using a generalized linear mixed model to examine the time × group interaction for between- and within-group changes. Due to multiple outcomes, the Benjamini-Hochberg procedure will be used to correct the raw *p*-values to minimize type I errors (false positives). The effect sizes will be reported using Cohen’s *d.* All models will adjust for age, gender, and BMI. For all tests, *p* ≤ 0.05 will be considered statistically significant.

## Results (Anticipated)

Research on the sleep habits and behaviors of shift workers and effective fatigue interventions in the mining industry is scarce. This methodology is unique as it is the first RCT study design involving 88 shift workers in a remote mining operation. It is designed and based on the results of the limited studies involving the sleep habits of shift workers and effective interventions (Legault et al., 2017; Barger et al., 2018; Choobineh et al., 2018), along with our industry knowledge of mining operations and the impact of sleep loss on shift workers. Although it is not possible to predict the exact outcomes of this study, there are several anticipated results.

### Compliance With Protocol

Of the 88 shift workers who will be enrolled in this study, we estimate a non-compliance rate of 20% with the study protocol. Potential reasons for non-compliance may include the removal of the wrist-activity monitors during a contact sport, showering, due to loss, illness, or leaving the organization. Data will be collected for 42-days and nights across all stages of the roster cycle (7 days/7 nights/7 off roster cycle), providing an estimated 40,656 sleep and alertness variables for analysis (14 nights per participant providing 1,232 nights of data).

### Sleep and Alertness

We anticipate that the duration of sleep achieved by participants will be less than the recommended 7–9 h (Hirshkowitz et al., 2015) for a healthy adult, and vary according to shift-type worked, that being, day shift or night shift as found in similar studies (Kecklund and Axelsson, 2016). Daytime sleep after the night shift is expected to be the shortest (6 h), whereas the longest sleep is expected following the day shift (6.5 h). The sleep duration of participants during their days off is expected to be highest (7 h) as they rest and recover from 14 days of continuous shiftwork (7 days/7 nights). Sleep loss resulting from the night shift is estimated to result in average alertness of <80%.

### Bodyweight, Alcohol Consumption, Sleep Problems and Disorders

#### Bodyweight

The prevalence of overweight and obesity in the general population is 67%, with 36% being overweight and 31% obese (Australian Bureau of Statistics, 2019). Similar levels of overweight and obesity, 40% and 28%, respectively, have been reported in the Australian mining industry (Street and Thomas, 2017). Therefore, we anticipate similar levels of overweight and obesity in this study.

#### Alcohol Consumption

Shiftwork that involves working nights is associated with excessive drinking (Richter et al., 2020). The Australian Institute of Health and Welfare estimates that 17% of the population consumes alcohol at hazardous levels, and one in four males are likely to drink at hazardous levels compared to one in ten females (Health and Welfare, 2019). In the Australian coal mining industry, 45.7% of males and 17.0% of females reported consuming alcohol at a hazardous or harmful level as measured by an AUDIT score of ≥8 (Tynan et al., 2017). Given this, and the predominately male shiftwork population reported here, we anticipate that alcohol consumption will be higher than the general population and similar to males in the coal mining industry.

#### Obstructive Sleep Apnea

The prevalence of OSA in the general population ranges from 9 to 38%, is higher amongst males (Senaratna et al., 2017), and is associated with obesity (Dong et al., 2020). We anticipate a higher prevalence in our study compared to the general population given the predominately male sample and expected high rates of obesity.

#### Shiftwork Disorder

The prevalence of shiftwork disorder in shiftwork populations varies between studies ranging from 23 to 63% (Vanttola et al., 2019). The symptoms of shiftwork disorder are similar to insomnia, including difficulty falling asleep and staying asleep, and excessive daytime sleepiness (Booker et al., 2018). We anticipate a similar prevalence, due to the roster schedule.

#### Insomnia

The prevalence of insomnia in the general population ranges from 5 to 33% (Reynolds et al., 2019). A study into the prevalence of insomnia amongst shift workers reported the prevalence of insomnia as 18.5% for night shift and 8.4% for day shift workers (Yong, 2017). We anticipate that the prevalence of shiftwork disorder will be similar.

#### Daytime Sleepiness

The prevalence of excessive daytime sleepiness in the general population is estimated to be 19% (Deloitte Access Economics, 2017). We anticipate the prevalence will be higher in our study, due to the circadian misalignment experienced by shift workers and our anticipated high prevalence of sleep apnea, insomnia, and shiftwork disorder, that may contribute to excessive daytime sleepiness.

### Advantages and Limitations

A strength of this study is the collection of longitudinal data over 42 nights from a representative sample (*n* = 88) of shift workers in a remote mining operation for the first time. This study also used objective measures including wrist-activity monitors and biomathematical modeling, and subjective measures including a survey instrument to capture sleep quality, quantity, alertness data, and assess the prevalence of risk for sleep disorders. It is important to note that a level of bias may have been introduced into this study as participants were self-selected, requiring a level of personal motivation.

A potential limitation of this study is the exclusion of PSG (in-laboratory assessment of measuring sleep) to determine the prevalence of sleep problems and disorders in a clinical setting. This is due to the logistical challenges of conducting PSG in a remote mining operation and the associated high costs that were estimated to be more than $50,000 for this study. Nevertheless, this study will provide valuable results on the prevalence of risk for sleep disorders in a mining operation. The high prevalence of risk for these sleep disorders and the potential adverse health and safety impact on shift workers may support a business case for mining operations to further investigate and invest in programs that incorporate PSG for the clinical diagnosis of sleep disorders.

A further limitation of this study was the ability of study participants to communicate between them and share knowledge of good sleep hygiene practices regardless of their assigned intervention. As participants reside in camp accommodation and spend 14 days and nights working, living, and socializing together, instructing participants not to communicate with each other and share knowledge was not feasible nor practical.

## Discussion

Peer-reviewed research is scarce on sleep and the efficacy of interventions to improve sleep quality, quantity, and alertness outcomes in shift workers in the mining industry. This protocol describes the design of an RCT that aims to (i) determine the efficacy of an intervention that comprised of a sleep education program and biofeedback through a smartphone app on sleep quality, quantity, and alertness, (ii) determine the prevalence of risk for a potential sleep disorder, and (iii) quantify and describe the sleep habits and behaviors of shift workers in a remote mining operation.

Shift workers frequently experience sleep loss due to the rotating design of a roster cycle. In remote mining operations, this typically requires shift workers to workdays, followed by nights, followed by a period of days off to rest. Such roster designs result in a circadian misalignment due to the requirement to sleep during the day following a night shift. This misalignment, along with long shift durations (>12 h), early morning start time, and the presence of undiagnosed and treated sleep disorders may result in sleep loss (Akerstedt and Wright, 2009).

Studies report night shift workers only achieve 5–6 h of sleep (following night shift) compared to day shift workers who achieve 6–7 h (Kecklund and Axelsson, 2016). This loss of sleep may result in fatigue and increase the risk of accidents within a mining operation (Halvani, 2009). Furthermore, chronic sleep loss over time (>10 years), can significantly increase the risk of health conditions including obesity, diabetes, cancer, cardiovascular disease, and mental health disorders (James et al., 2017). Therefore, high-quality studies are necessary to provide an evidence base for effective interventions to improve sleep outcomes of shift workers and reduce fatigue risk within mining operations.

### Practical Considerations

This study will be conducted in a remote WA mining operation under a business as usual model. A collaborative partnership with the WA mining operation has been identified as a critical success factor in deploying this study protocol as it will assist in identifying solutions to overcome any barriers that arise. The following practical considerations for future research in mining operations have been identified:

(1) Mine sites are often subjected to unforeseen events including incidents, equipment breakdowns, or hazardous weather conditions, including tropical cyclones or electrical storms. The occurrence of these events can negatively impact productivity, making them a priority to manage for the mine site, and potentially interfering with research activities.
(2) Study data collection methods should be practical and appropriate to optimize the collection of valid data. The use of technology such as wrist-activity monitors requires participants to be well informed and correctly use the device for the study duration (e.g., the requirement to wear the device 24 h, 7 days a week for the 42-day study). Incorrect device use may result in data loss. Online platforms such as Qualtrics^TM^ should be considered for the administration of survey instruments. Such platforms allow participants to enter their responses through their mobile phone devices, providing a practical and time-efficient alternative to a computer or paper-based data collection method. The limited physical space in mine site operational areas is often not designed for computers or paper-based work.
(3) Societal and public health factors, such as the COVID-19 global pandemic have the potential to interfere with the study protocol due to changes to roster cycles, and restriction to site access potentially limiting face-to-face contact with participants. Although often outside the control of the researcher, such factors should be considered as part of the initial risk assessment to minimize the impact.

## Conclusion

The findings from our study will contribute to the current understanding of sleep and alertness behaviors, the prevalence of risk for sleep disorders, and the efficacy of interventions to improve sleep quantity, quality, and alertness of shift workers in remote mining operations. The study findings have the potential to inform policy and business practice on how to manage fatigue risk effectively. If the use of a sleep education program and biofeedback through smartphone apps is found to be effective in improving sleep quantity, quality, and alertness, these interventions may be a critical component of an organization’s broader systematic approach to managing fatigue risk. The study protocol described in this paper may be applied to other industries including oil and gas, aviation, rail, and healthcare for the assessment of similar fatigue interventions and to determine the potential prevalence of sleep problems and disorders.

## Data Availability Statement

As this is a methodology paper there are currently no data sets available. Under agreement with the participating mining company data will be held by Edith Cowan University only. Requests to access the datasets should be directed to GM, g.maisey@ecu.edu.au.

## Ethics Statement

The studies involving human participants were reviewed and approved by Edith Cowan University Human Research Ethics (approval number: 2019-00813-MAISEY). The patients/participants provided their written informed consent to participate in this study. Written informed consent was obtained from the individual(s) for the publication of any potentially identifiable images or data included in this article.

## Author Contributions

GM designed the study, collected the data, and drafted the manuscript. MC, AD, ID, and JL assisted in designing the study and editing the manuscript. JL provided the support with statistical analysis. All authors contributed to the article and approved the submitted version.

## Conflict of Interest

ID is the Chair of the Scientific Advisory Board for Fatigue Science, Canada, and received no financial or other incentives related to this research project. GM is employed on a casual basis by Melius Consulting Pty Ltd., for which ID is the Director and Chief Adviser. The remaining authors declare that the research was conducted in the absence of any commercial or financial relationships that could be construed as a potential conflict of interest.
