# Supplementary material for: The Sleep of Shift Workers in a Remote Mining Operation: Methodology for a Randomized Control Trial to Determine Evidence-Based Interventions
Source: Front Neurosci. 2021 Jan 7;14:579668. doi: 10.3389/fnins.2020.579668 (PMC7817759; doi:10.3389/fnins.2020.579668)
Supplement: Supplementary file 1 [file Table_1.DOCX]

Sleep Hygiene Quiz

# The recommended amount of sleep for adults (aged 18-64 yrs.) to maintain optimum health and wellbeing is?

- 1. 5-6 hrs.
  2. 7-9 hrs.
  3. 9-10 hrs.
  4. 10+ hrs.

1. Our bodies sleep-wake cycle is controlled by the suprachiasmatic nucleus (SCN), which is regulated by light?
   1. True
   2. False
2. According to science, consuming alcohol close to bedtime
   1. Helps me get to sleep faster
   2. Helps me get a better night’s sleep
   3. Helps me get to sleep faster, but disrupts my sleep later in the night
   4. Helps me to wake-up refreshed the next morning
3. Blue light and stimulating activity from your iPhone, iPad or computer may affect your sleep and should be avoided directly before bedtime
   1. True
   2. False
4. Caffeine should be avoided at least 4 hrs. before bedtime for a good night’s sleep.
   1. True
   2. False
5. A good bedtime routine is an important strategy in helping you fall asleep and getting a good night’s sleep. Which of the following are not recommended as part of your bedtime routine (select multiple responses)?
   1. Reading a novel
   2. Logging onto social media, i.e. Facebook, Instagram
   3. Exercising
   4. Taking a shower (warm or cold)
   5. Drinking coffee
6. Regular exercise can improve your sleep and increase your alertness during the day but should be avoided
   1. First thing in the morning (i.e. before 6 am)
   2. Early afternoon
   3. 3-4 hrs. before bedtime
7. If you cannot get to sleep within 20 mins of going to bed, you should get up and do something relaxing until you feel sleepy then try again?
8. True
9. False
10. When on nightshift you should aim for at least 6 hrs. sleep?
11. True
12. False
13. You will know if you have a microsleep?
    1. True
    2. False
14. A 90-minute nap (one complete sleep cycle) before the night shift can improve alertness and performance by about 30%.
    1. True
    2. False
15. At what times is it easier to fall asleep (circle all correct answers)?
    1. Early afternoon, 1–3 pm.
    2. In the morning, around 9–10 am.
    3. In the early evening, around 6–9 pm.
    4. Pre-dawn hours around 3–4 am.
    5. None of the above

Thank you for completing this quiz

**Answers:** Q1(b); Q2(a); Q3(c); Q4(a); Q5(a); Q6(b)(c)(e); Q7(c); Q8(a); Q9(a); Q10(b); Q11(a); Q12(a)(d)
